# Coagulation markers as independent predictors of prostate cancer aggressiveness: a retrospective cohort study

**DOI:** 10.1038/s41598-023-43427-w

**Published:** 2023-09-26

**Authors:** Xu Lei, Tengfei Zhang, Zhixuan Deng, Tao Jiang, Yang Hu, Ning Yang

**Affiliations:** The Second Affiliated Hospital of University of South China, Hengyang, 421001 Hunan China

**Keywords:** Cancer, Diseases, Oncology, Urology

## Abstract

Coagulation system activation is commonly observed in tumor patients, including prostate cancer (PCa), with coagulation markers proposed as potential prognostic indicators for cancer severity. However, the correlation between these markers and clinicopathological features in PCa remains unclear. Thus, this study investigates the association between comprehensive coagulation markers and clinicopathological characteristics in PCa patients. A retrospective evaluation of 162 PCa patients diagnosed and categorized into low-intermediate-risk or high-risk groups based on clinical and pathological features was conducted. Coagulation markers, including fibrinogen (FIB), d-dimer (DD), activated partial thromboplastin time (APTT), prothrombin time (PT), prothrombin activity (PTA), thrombin time (TT), platelet count (PLT), and international normalized ratio (INR), were assessed. Univariate and multivariate logistic regression analyses were performed to determine associations with clinicopathological features. FIB and DD were confirmed as independent factors associated with high-risk PCa. Furthermore, FIB and DD levels showed significant positive correlations with clinical parameters, including PSA levels, ISUP grade, T stage, N stage, and M stage. Our findings suggest that FIB and DD hold promise as independent prognostic biomarkers for risk stratification in PCa. These coagulation markers may aid in assessing PCa severity and guiding personalized treatment strategies.

## Introduction

Prostate cancer (PCa) represents a critical global health challenge. It ranks as the fourth most common malignancy worldwide and exhibits the second-highest frequency among male neoplasms. The morbidity and mortality of PCa have exhibited a concerning upward trend globally in recent years^1^. Currently, the pathological classification, Gleason grading system, and clinicopathological staging are widely employed in clinical settings to assess the severity and prognostic implications of PCa. However, the majority of the aforementioned parameters necessitate acquisition via prostate biopsy. Regrettably, the positivity rate of prostate biopsy is often suboptimal and may be accompanied by hemorrhage, infectious complications, and other hazards^2^. Thus, how to accurately identify the severity of PCa before prostate biopsy is an essential direction of current research, which would be beneficial in PCa risk stratification.

A multitude of investigations has established a close relationship between abnormal coagulation function and the advancement of cancer^3^. In the presence of tumors, coagulation-related mechanisms within the tumor stroma and surrounding microenvironment can become activated. The degree of activation has been associated with tumor cell proliferation and metastasis^4–7^. Abnormal activation of the coagulation system can expedite malignant proliferation, invasion, and metastasis through the augmentation of tumor vascular adhesion, promotion of tumor growth and angiogenesis, and facilitation of the tumor's immune system evasion^8–10^. The intricate interplay between malignant tumors and the coagulation system fosters a hypercoagulable state in tumor patients, and varying levels of coagulation status may delineate the underlying biological attributes of tumors. Consequently, peripheral blood coagulation parameters, which mirror the systemic coagulation state, hold promise as potential indicators for tumor risk stratification. The levels of coagulation markers have been found to be significantly correlated with the clinicopathological features of malignancies, such as gastric cancer, breast cancer, and lung cancer^11–13^. However, a paucity of research has investigated the correlation between coagulation markers and the clinicopathological features of PCa. Moreover, current studies have concentrated on individual coagulation markers, such as activated partial thromboplastin time (APTT) or prothrombin time (PT), while disregarding other pertinent markers. Consequently, the connection between coagulation markers and the risk of PCa severity remains inadequately and incompletely evaluated.

The present study evaluated a comprehensive panel of coagulation markers, encompassing APTT, PT, fibrinogen (FIB), d-dimer (DD), prothrombin activity (PTA), thrombin time (TT), platelet count (PLT), and international normalized ratio (INR). These coagulation parameters hold a predominant position as the prevailing choice in clinical practice. Through the comprehensive evaluation of the interplay between coagulation markers and clinicopathological features of PCa, our study endeavors to elucidate the potential value of coagulation markers in the risk stratification of PCa.

## Methods

### Patients

A total of 876 patients who underwent transrectal ultrasound-guided prostate biopsies and received a pathological diagnosis of PCa at the Second Affiliated Hospital of University of South China were collected between January 2017 and December 2022.

Rigorous inclusion criteria were established to ensure the dependability and validity of the findings. The following were the inclusion criteria: (1) patients with complete case data during diagnosis and treatment, (2) patients diagnosed with PCa by pathological examination, (3) patients with no concurrent or prior diagnosis or treatment of other primary malignancies, (4) patients without radiotherapy, chemotherapy and endocrine therapy before treatment, (5) patients without autoimmune system diseases, liver diseases, blood system diseases, infectious diseases, cardiovascular and cerebrovascular accidents and other diseases that may affect the level of coagulation parameters, (6) patients who have not received procoagulant or anticoagulant therapy 8 weeks before the visit, and (7) patients with no history of major trauma or major surgery 8 weeks prior to presentation. These criteria were carefully chosen to guarantee that the study participants had a similar medical background and that their coagulation parameters were unaffected by external factors. The application of these criteria would provide a more accurate and reliable basis for subsequent research analyses.

**Figure 1:**
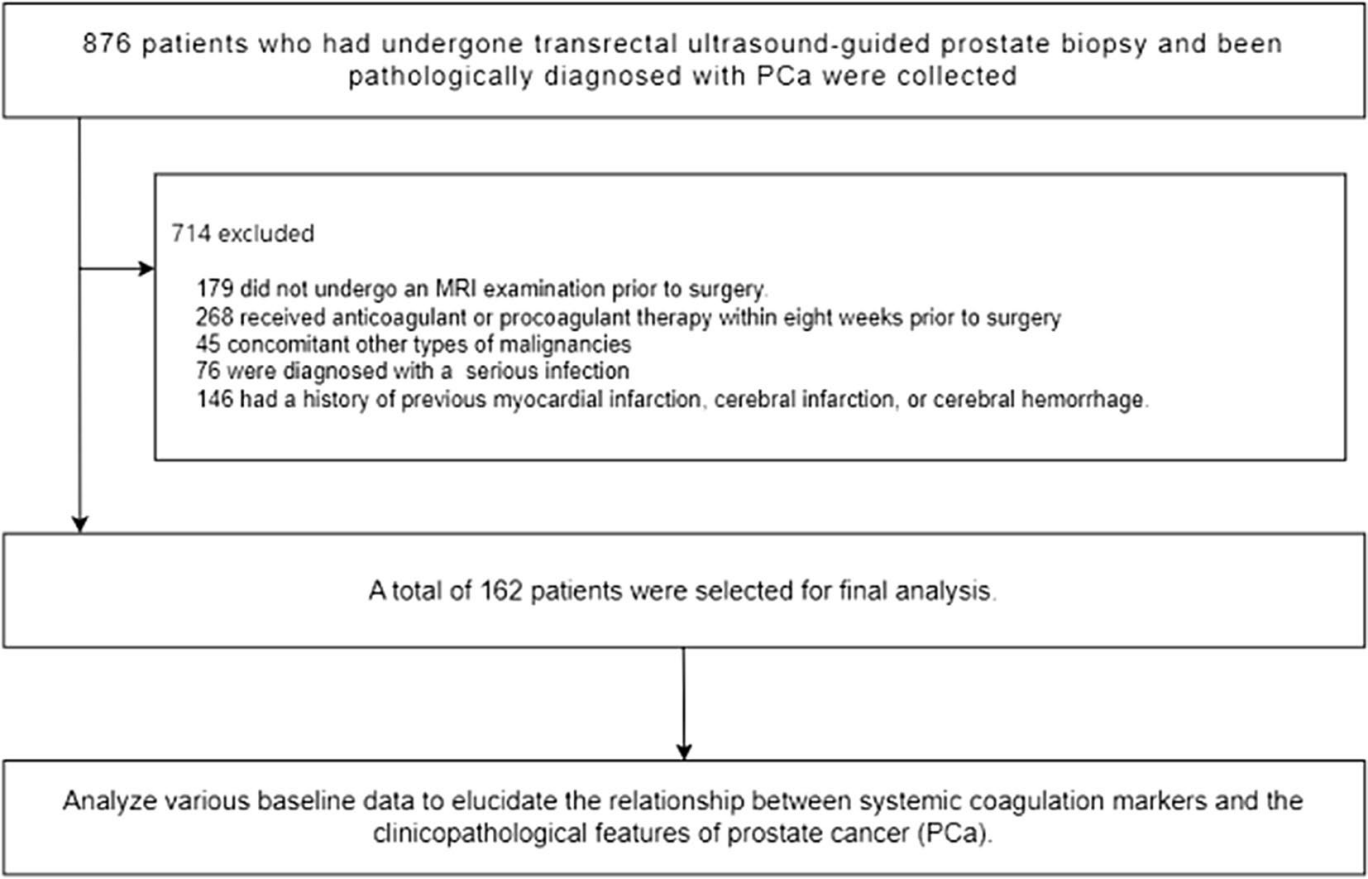
Flowchart of the patient selection process.

Following the strict application of our inclusion criteria, 162 patients with PCa were selected for inclusion in this study. We conducted a retrospective analysis of the patient's baseline characteristics, preoperative laboratory and imaging tests, and postoperative pathological findings (Fig. 1).

This study adheres to the guidelines outlined by the Strengthening the Reporting of Cohort Studies in Surgery (STROCSS) initiative.

### Data collection

We retrospectively gathered the clinicopathological data of 162 patients diagnosed with PCa. The dataset encompassed pertinent clinical characteristics such as the preoperative laboratory test, imaging results, and postoperative pathological results. Furthermore, baseline characteristics such as age, smoking history, diabetes, and hypertension were included in the analysis (Table 1). All relevant clinicopathological data were obtained from medical records, and all prostate biopsy specimens were sent to the pathology department for diagnostic evaluation. The prostate cancer pathology grade was performed based on the 2014 International Society of Urological Pathology (ISUP) classification system^14^. The clinical stage of PCa was determined by magnetic resonance imaging (MRI) and adhered to the 2017 edition of the American Joint Committee on Cancer (AJCC) TNM staging system for malignant tumors^15^.

**Table 1:** Clinical participant clinicopathological data.

All patients (N = 162)	
Age, years	
Mean (SD)	71.4 (7.0)
Median (Q1–Q3)	72 (66–77)
Smoking history, n (%)	
Exist	83 (51.2)
N/A	79 (48.8)
Hypertension, n (%)	
Exist	56 (34.6)
N/A	106 (65.4)
Diabetes mellitus, n (%)	
Exist	17 (10.5)
N/A	145 (89.5)
T stage, n (%)	
T1	35 (21.6)
T2	36 (22.2)
T3	46 (28.4)
T4	45 (27.8)
N stage, n (%)	
N0	101 (62.3)
N1	61 (37.7)
M stage, n (%)	
M0	105 (64.8)
M1	57 (35.2)
ISUP grade, n (%)	
1	36 (22.2)
2	20 (12.3)
3	19 (11.7)
4	27 (16.7)
5	60 (37.1)
PSA (ng/mL)	
Mean (SD)	54.9 (42.4)
Median (Q1–Q3)	39.1 (25.1–84.0)
PLT (×10^9^/L)	
Mean (SD)	196.4 (65.7)
Median (Q1–Q3)	191 (155–230)
DD (mg/L)	
Mean (SD)	1.2 (2.5)
Median (Q1–Q3)	0.6 (0.4–1.1)
FIB (g/L)	
Mean (SD)	3.7 (1.3)
Median (Q1–Q3)	3.7 (2.8–4.5)
PT (s)	
Mean (SD)	12.5 (1.0)
Median (Q1–Q3)	12.5 (12.0–13.0)
PTA (%)	
Mean (SD)	106.1 (17.7)
Median (Q1–Q3)	100 (100–116.3)
INR	
Mean (SD)	0.98 (0.09)
Median (Q1–Q3)	0.99 (0.92–1.00)
APTT (s)	
Mean (SD)	36.5 (4.2)
Median (Q1–Q3)	36.1 (33.7–39.4)
TT (s)	
Mean (SD)	16.7 (2.0)
Median (Q1–Q3)	16.4 (15.6–17.4)

Following the ethical standards outlined in the Declaration of Helsinki, the Ethics Committee of the Second Affiliated Hospital of University of South China approved this study. Informed consent was obtained from all participants prior to their inclusion in the study.

### Statistical analysis

The normality of the distribution of quantitative variables was assessed using the Shapiro–Wilk test. For variables with a normal distribution, an independent-sample T-test or one-way ANOVA was applied, and descriptive statistics were reported as mean ± standard deviation (SD). For variables with a non-normal distribution, the Mann–Whitney U test or Kruskal–Wallis H test was applied and presented as medians (Q1–Q3). Categorical variables were presented as frequencies and percentages and analyzed using the chi-square test. All PCa participants were previously categorized into low-intermediate-risk and high-risk groups, as previously described^16^. A comparison was conducted between the two groups with regard to differences in coagulation parameters and clinicopathological features. The FIB levels were divided into tertiles: T1 (< 2.99 g/L, 33.3rd percentile; n = 53), T2 (2.99–4.22 g/L, 33.3–66.7th percentile; n = 54), and T3 (≥ 4.23 g/L, 66.7–100th percentile; n = 55). We assessed the clinicopathological and coagulation characteristics in each FIB-level group. Similarly, the DD levels were divided into tertiles: G1 (< 0.48 mg/L, 33.3rd percentile; n = 53), G2 (0.48–0.78 mg/L, 33.3–66.7th percentile; n = 53), and G3 (≥ 0.79 mg/L, 66.7–100th percentile; n = 56). We then analyzed the clinicopathological and coagulation characteristics in each DD-level group. A univariate logistic regression analysis was employed to evaluate the correlations between coagulation markers and high-risk PCa. Multivariate regression analysis was used to identify independent factors associated with high-risk PCa. Statistical analyses were conducted using SPSS software (version 25.0; SPSS Inc., Chicago, IL, USA), and *p* < 0.05 was considered statistically significant.

## Results

### Baseline patient characteristics

Table 1 provides a summary of the baseline characteristics of the study participants. A total of 162 patients were enrolled, with a mean age of 71 years (range 56–91 years). Among these patients, 83 (51.2%) had a history of smoking, 56 (34.6%) had a history of hypertension, and 17 (10.5%) had a history of diabetes mellitus. The clinical stage of PCa was determined by MRI and adhered to the 2017 edition of the American Joint Committee on Cancer TNM staging system for malignant tumors^15^. The majority of the patients in the study were diagnosed with advanced-stage disease, as evidenced by the classification of 35 patients (21.6%) as T1 stage, 36 patients (22.2%) as T2 stage, 46 patients (28.4%) as T3 stage, and 45 patients (27.8%) as T4 stage. Additionally, lymph node metastasis was detected in 61 patients (37.7%), while 57 patients (35.7%) had distant metastasis. The ISUP grade was used to assess the tumor grade of patients, with 36 patients (22.2%) classified as grade 1, 20 patients (12.3%) classified as grade 2, 19 patients (11.7%) classified as grade 3, 27 patients (16.7%) classified as grade 4, and 60 patients (37.1%) classified as grade 5. In terms of laboratory parameters, the median PSA level was 39.1 ng/mL (interquartile range [IQR] 25.1–84.0 ng/mL). The median PLT was 191 × 10^9^/L (IQR 155–230 × 10^9^/L), the median DD level was 0.6 mg/L (IQR 0.4–1.1 mg/L), and the median FIB level was 3.7 g/L (IQR 2.8–4.5 g/L). The median PT was 12.5 s (IQR 12.0–13.0 s), the median PTA was 100% (IQR 100–116.3%), the median INR was 0.99 (IQR 0.92–1.00), the median APTT was 36.1 s (IQR 33.7–39.4 s), and the median TT was 16.4 s (IQR 15.6–17.4 s).

### Comparison of clinicopathological characteristics and coagulation markers between low-intermediate-risk and high-risk PCa patients

**Figure 2:**
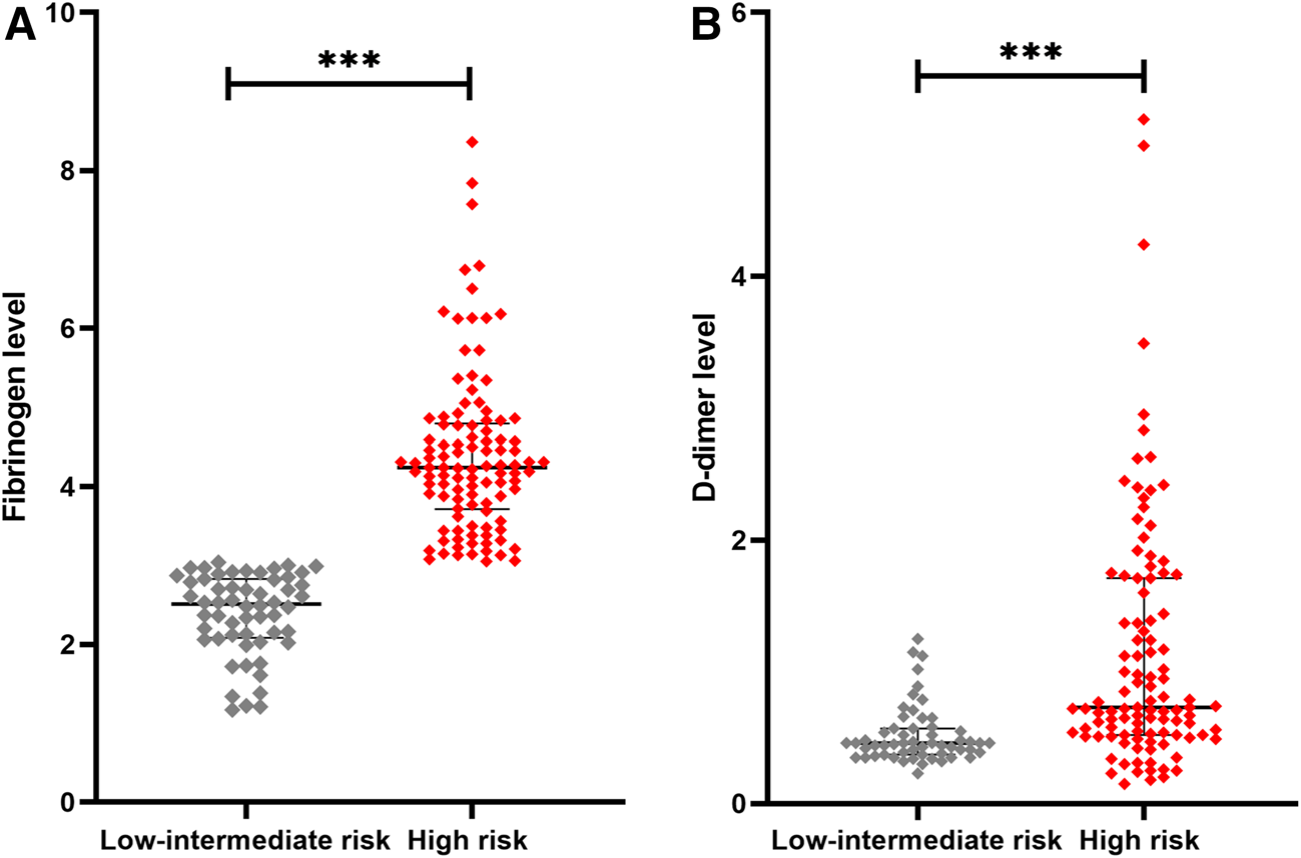
Comparison of coagulation markers in low-intermediate-risk and high-risk groups of patients. (**A**) High-risk PCa is related to fibrinogen level. (**B**) High-risk PCa is related to d-dimer level. ***p < 0.001.

In the present study, patients were stratified into two distinct categories of risk: the low-intermediate-risk group (n = 56, 34.57%), and the high-risk group (n = 106, 65.43%). Our study compared the clinicopathological characteristics and coagulation markers between these two groups. The findings of the present investigation demonstrated significant statistical dissimilarities in PSA levels, ISUP grade, T stage, N stage, and M stage between the two study cohorts (*p* < 0.001 for all), while no noteworthy distinctions in age, smoking history, hypertension, or diabetes were detected between the groups (*p* > 0.05 for all) (Table 2). Notably, the levels of FIB and DD were significantly elevated in the high-risk cohort in contrast to the low-risk group (*p* < 0.001 for both), while PLT, PT, PTA, APTT, INR, and TT were found to be similar between the two groups (*p* > 0.05 for all) (Fig. 2, Table 2).

**Table 2:** A descriptive analysis of the baseline characteristics of study participants stratified by the risk of PCa.

	Low-intermediate risk (n = 56)	High risk (n = 106)	*p* value
Demographic characteristics			
Age (years)	70.79 ± 6.34	71.75 ± 7.28	0.406
Smoking history, n (%)			0.919
Exist	29 (51.8)	54 (50.9)	
N/A	27 (48.2)	52 (49.1)	
Hypertension, n (%)			0.568
Exist	21 (37.5)	35 (33.0)	
N/A	35 (62.5)	71 (67.0)	
Diabetes mellitus, n (%)			0.637
Exist	5 (8.9)	12 (11.3)	
N/A	51 (91.1)	94 (88.7)	
Clinicopathological characteristics			
PSA (ng/mL)	21.92 (6.03–26.54)	70.29 (39.11–97.90)	** < 0.001**
T stage, n (%)			** < 0.001**
T1	34 (60.7)	1 (0.9)	
T2	22 (39.3)	14 (13.2)	
T3	0	46 (43.4)	
T4	0	45 (42.5)	
N stage, n (%)			** < 0.001**
N0	56 (100)	45 (42.5)	
N1	0	61 (57.5)	
M stage, n (%)			** < 0.001**
M0	56 (100)	49 (46.2)	
M1	0	57 (53.8)	
ISUP grade, n (%)			** < 0.001**
1	35 (62.5)	1 (0.9)	
2	17 (30.4)	3 (2.8)	
3	4 (7.1)	15 (14.2)	
4	0	27 (25.5)	
5	0	60 (56.6)	
Coagulation markers			
PLT (×10^9^/L)	188 (152–217)	193 (155–231)	0.790
FIB (g/L)	2.62 ± 0.76	4.23 (3.47–4.79)	** < 0.001**
DD (mg/L)	0.46 (0.37–0.57)	0.73 (0.52–1.72)	** < 0.001**
PT (s)	12.4 (12.0–13.2)	12.5 (11.9–13.0)	0.938
PTA (%)	100 (94–112)	103.5 (100.0–117.5)	0.063
APTT (s)	36.58 ± 4.31	36.52 ± 4.13	0.924
TT (s)	16.55 (15.8–17.5)	16.30 (15.3–17.3)	0.160
INR	1.00 (0.94–1.03)	0.98 (0.91–1.00)	0.066

Significant values are in bold.

### Plasma FIB levels correlate with PCa severity

We investigated the association between plasma FIB levels and a range of clinical and pathological characteristics of PCa patients, using Spearman's rank correlation test. Our findings demonstrated a significant positive association between plasma FIB levels and various indicators of PCa severity, including PSA levels (r = 0.583, *p* < 0.001), ISUP grade (r = 0.515, *p* < 0.001), T stage (r = 0.682, *p* < 0.001), N stage (r = 0.463, *p* < 0.001), and M stage (r = 0.507, *p* < 0.001) (Table 3).

**Table 3:** Correlation between FIB and clinicopathological characteristics of PCa.

	PSA		ISUP grade,		T stage		N stage		M stage	
	r	*p* value	r	*p* value	r	*p* value	r	*p* value	r	*p* value
FIB	0.583	< 0.001	0.515	< 0.001	0.682	< 0.001	0.463	< 0.001	0.507	< 0.001

**Figure 3:**
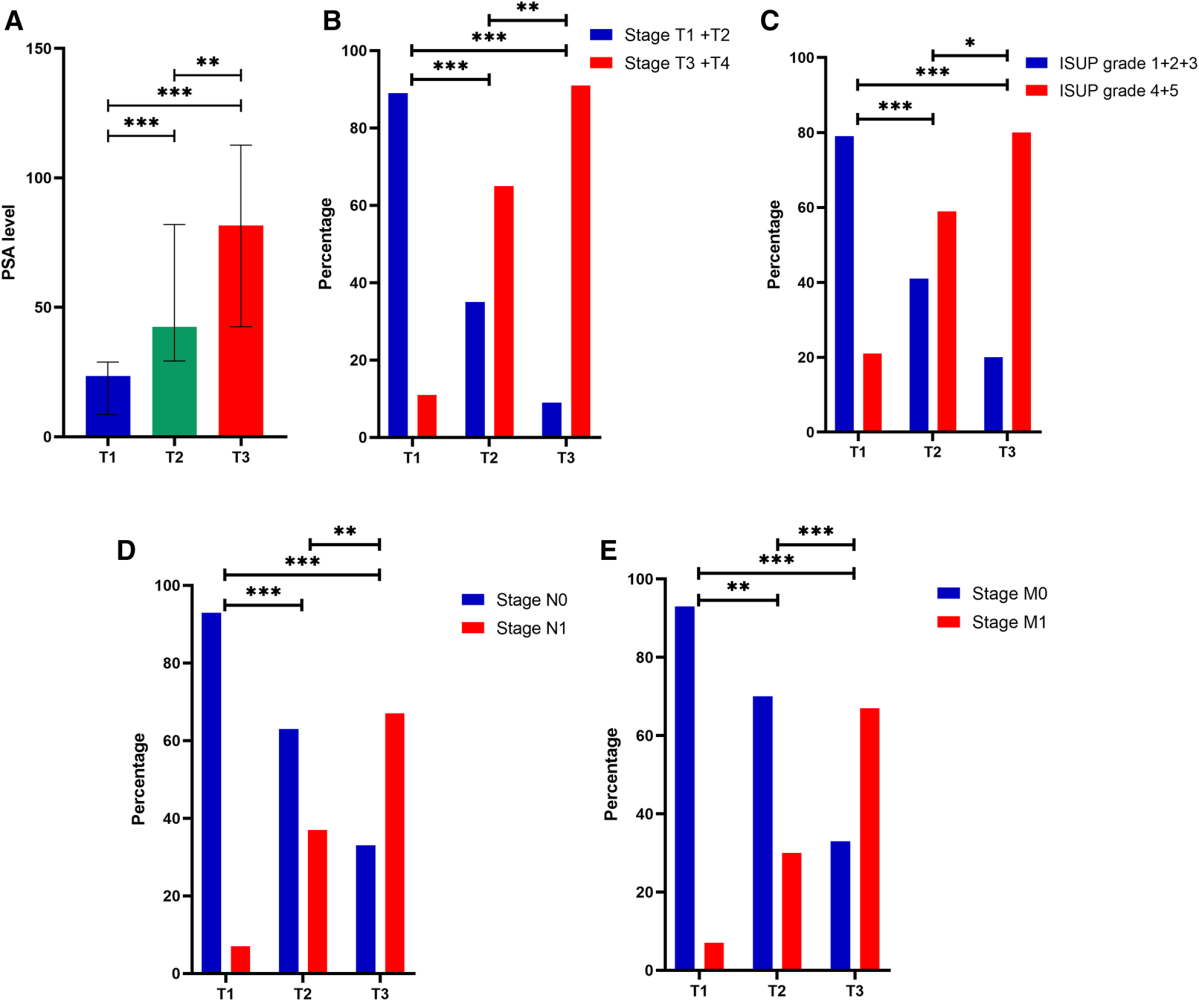
Comparison of clinicopathological characteristics in patients stratified into tertiles by plasma fibrinogen levels. (**A**) PSA levels in PCa patients are associated with fibrinogen levels. (**B**) The tumor T stage of patients with PCa is associated with fibrinogen levels. (**C**) The ISUP grade of patients with PCa is associated with fibrinogen levels. (**D**) The tumor N stage of patients with PCa is associated with fibrinogen levels. (**E**) The tumor M stage of patients with PCa is associated with fibrinogen levels. *0.01 ≤ *p* < 0.05, **0.001 ≤ P < 0.01, ***p < 0.001.

Then, patients with PCa were categorized into three groups according to tertiles of plasma FIB levels, as previously described. Statistical analysis revealed significant differences in PSA levels, ISUP grade, T stage, regional lymph node metastasis, and distant metastasis among the three groups (*p* < 0.001 for all), while no significant differences were found in age, smoking history, hypertension, or diabetes among the three groups (*p* > 0.05 for all) (Table 4). Specifically, there was a statistically significant trend of increasing PSA levels, late-stage (stage T3 + T4) percentages, and high-grade (ISUP grade 4 + 5) percentages with increasing FIB levels. Moreover, a significant increase in the proportion of patients with regional lymph node metastasis and distant metastasis was observed with increasing FIB levels (Fig. 3). In relation to coagulation markers, significant differences in DD, PTA, TT, and INR were observed among the three FIB groups (*p* < 0.001, *p* = 0.001, *p* = 0.006, *p* = 0.002). However, no significant differences were found in PLT, PT, and APTT among the three groups (*p* > 0.05 for all) (Table 4).

**Table 4:** Clinical characteristics of subjects categorized by tertiles of plasma FIB levels.

	T1 (< 2.99 g/L, n = 53)	T2 (2.99–4.22 g/L, n = 54)	T3 (≥ 4.23 g/L, n = 55)	*p* value
Demographic characteristics				
Age (years)	71.06 ± 6.78	72.59 ± 6.63	70.60 ± 7.42	0.297
Smoking history, n (%)				0.663
Exist	28 (52.8)	25 (46.3)	30 (54.5)	
N/A	25 (47.2)	29 (53.7)	25 (45.5)	
Hypertension, n (%)				0.113
Exist	18 (34.0)	24 (44.4)	14 (25.5)	
N/A	35 (66.0)	30 (55.6)	41 (74.5)	
Diabetes mellitus, n (%)				0.916
Exist	6 (11.3)	6 (11.1)	5 (9.1)	
N/A	47 (88.7)	48 (88.9)	50 (90.9)	
Clinicopathological characteristics				
PSA (ng/mL)	23.45 (8.53–28.87)	42.41 (29.29–81.99)	79.91 ± 42.78	** < 0.001**
T stage, n (%)				** < 0.001**
T1	28 (52.8)	5 (9.3)	2 (3.6)	
T2	19 (35.8)	14 (25.9)	3 (5.5)	
T3	2 (3.8)	26 (48.1)	18 (32.7)	
T4	4 (7.6)	9 (16.7)	32 (58.2)	
N stage, n (%)				** < 0.001**
N0	49 (92.5)	34 (63.0)	18 (32.7)	
N1	4 (7.5)	20 (37.0)	37 (67.3)	
M stage, n (%)				** < 0.001**
M0	49 (92.5)	38 (70.4)	18 (32.7)	
M1	4 (7.5)	16 (29.6)	37 (67.3)	
ISUP grade, n (%)				** < 0.001**
1	24 (45.3)	9 (16.7)	3 (5.5)	
2	14 (26.4)	4 (7.4)	2 (3.6)	
3	4 (7.5)	9 (16.7)	6 (10.9)	
4	3 (5.7)	14 (25.9)	10 (18.2)	
5	8 (15.1)	18 (33.3)	34 (61.8)	
Coagulation markers				
PLT (×10^9^/L)	186.91 ± 52.64	185 (149–216)	204 (163–255)	0.083
DD (mg/L)	0.46(0.37–0.65)	0.55 (0.43–0.79)	1.12 (0.71–1.75)	** < 0.001**
PT (s)	12.46 ± 0.95	12.30 (11.90–12.83)	12.70 (12.00–13.30)	0.362
PTA (%)	100 (96–111)	112.24 ± 15.86	100 (97–110)	**0.001**
APTT (s)	36.34 ± 4.61	36.30 ± 4.16	36.98 ± 3.79	0.638
TT (s)	16.9 (16.0–17.7)	16.1 (15.1–16.9)	16.5 (15.2–17.8)	**0.006**
INR	1.00 (0.95–1.02)	0.95 (0.90–0.99)	1.00 (0.94–1.01)	**0.002**

Significant values are in bold.

### Plasma DD levels correlate with PCa severity

Based on our analysis using Spearman's rank correlation test, a significant positive correlation was observed between plasma DD levels and various clinical and pathological characteristics of PCa patients, including PSA levels (r = 0.457, *p* < 0.001), ISUP grade (r = 0.393, *p* < 0.001), T stage (r = 0.461, *p* < 0.001), N stage (r = 0.356, *p* < 0.001), and M stage (r = 0.409, *p* < 0.001) (Table 5).

**Table 5:** Correlation between DD and clinicopathological characteristics of PCa.

	PSA		ISUP grade,		T stage		N stage		M stage	
	r	*p* value	r	*p* value	r	*p* value	r	*p* value	r	*p* value
DD	0.457	< 0.001	0.393	< 0.001	0.461	< 0.001	0.356	< 0.001	0.409	< 0.001

**Figure 4:**
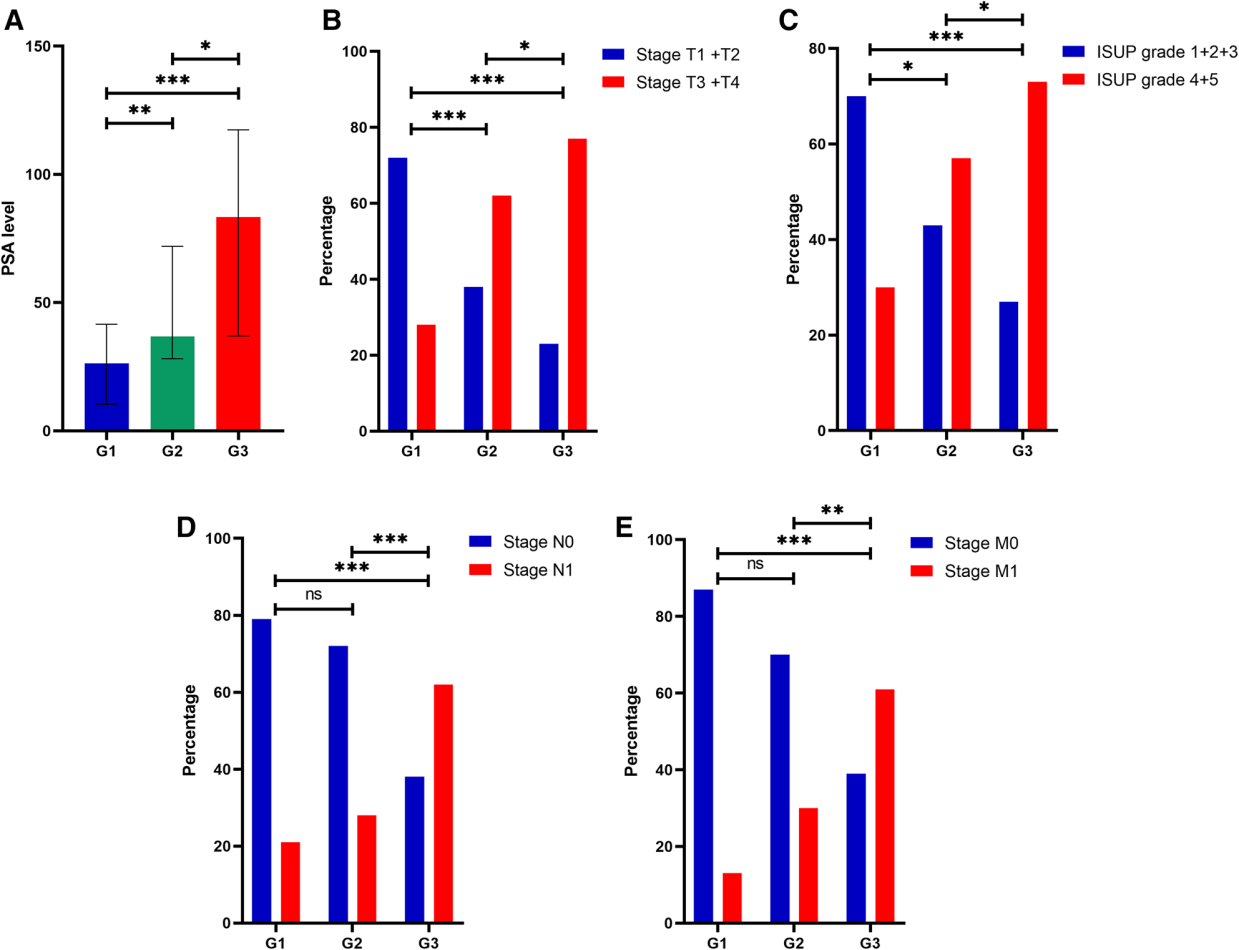
Comparison of clinicopathological characteristics in patients stratified into tertiles by plasma d-dimer levels. (**A**) PSA levels in PCa patients are associated with d-dimer levels. (**B**) The tumor T stage of patients with PCa is associated with d-dimer levels. (**C**) The ISUP grade of patients with PCa is associated with d-dimer levels. (**D**) The tumor N stage of patients with PCa is associated with d-dimer levels. (**E**) The tumor M stage of patients with PCa is associated with d-dimer levels. * 0.01 ≤ *p* < 0.05, ** 0.001 ≤ P < 0.01, ***p < 0.001, ns *p* > 0.05.

Subsequently, patients diagnosed with PCa were classified into three groups according to the tertiles of DD levels, as described previously. Statistical analysis revealed significant differences in PSA levels, ISUP grade, T stage, regional lymph node metastasis, and distant metastasis among the three groups (*p* < 0.001 for all), while there were no significant differences observed among the three groups in terms of age, smoking history, hypertension, or diabetes (*p* > 0.05 for all) (Table 6). Notably, there was a significant trend of increasing PSA levels, late-stage (stage T3 + T4) percentages, and high-grade (ISUP grade 4 + 5) percentages with increasing DD levels. Furthermore, our analysis demonstrated a significant rise in the proportion of patients with regional lymph node metastasis and distant metastasis in correlation with increasing DD levels (Fig. 4). In terms of coagulation markers, our findings suggest significant differences in FIB across the three DD groups (*p* < 0.001). Nonetheless, no significant differences were noted among the three groups in terms of PLT, PT, PTA, APTT, TT, and INR (*p* > 0.05 for all) (Table 6).

**Table 6:** Clinical characteristics of subjects categorized by tertiles of plasma DD levels.

	G1 (< 0.48 mg/L, n = 53)	G2 (0.48–0.78 mg/L, n = 53)	G3 (≥ 0.79 mg/L, n = 56)	*p* value
Demographic characteristics				
Age (years)	70.75 ± 7.30	71.40 ± 5.88	72.05 ± 7.62	0.625
Smoking history, n (%)				0.181
Exist	26 (49.1)	23 (43.4)	34 (60.7)	
N/A	27 (50.9)	30 (56.6)	22 (39.3)	
Hypertension, n (%)				0.311
Exist	21 (39.6)	20 (37.7)	15 (26.8)	
N/A	32 (60.4)	33 (62.3)	41 (73.2)	
Diabetes mellitus, n (%)				0.681
Exist	6 (11.3)	4 (7.5)	7 (12.5)	
N/A	47 (88.7)	49 (92.5)	49 (87.5)	
Clinicopathological characteristics				
PSA (ng/mL)	26.32 (10.32–41.55)	37.95 (28.60–71.99)	83.40 (37.02–117.35)	** < 0.001**
T stage, n (%)				** < 0.001**
T1	22 (41.5)	7 (13.2)	6 (10.7)	
T2	16 (30.2)	13 (24.5)	7 (12.5)	
T3	10 (18.9)	23 (43.4)	13 (23.2)	
T4	5 (9.4)	10 (18.9)	30 (53.6)	
N stage, n (%)				** < 0.001**
N0	42 (79.2)	38 (71.7)	21 (37.5)	
N1	11 (20.8)	15 (28.3)	35 (62.5)	
M stage, n (%)				** < 0.001**
M0	46 (86.8)	37 (69.8)	22 (39.3)	
M1	7 (13.2)	16 (30.2)	34 (60.7)	
ISUP grade, n (%)				** < 0.001**
1	23 (43.4)	7 (13.2)	6 (10.7)	
2	12 (22.6)	6 (11.3)	2 (3.6)	
3	2 (3.8)	10 (18.9)	7 (12.5)	
4	5 (9.4)	11 (20.8)	11 (19.6)	
5	11 (20.8)	19 (35.8)	30 (53.6)	
Coagulation markers				
PLT (×10^9^/L)	189.51 ± 52.95	193.0 (156.5–229.0)	191.5 (153.0–242.8)	0.861
FIB (g/L)	2.81 ± 0.82	3.83 ± 1.06	4.45 ± 1.41	** < 0.001**
PT (s)	12.48 ± 0.79	12.40 (11.90–13.00)	12.65 (11.93–13.38)	0.489
PTA (%)	100 (99.5–119.5)	101 (100–118)	100 (95.3–110.8)	0.155
APTT (s)	36.91 ± 4.37	36.34 ± 3.52	36.38 ± 4.59	0.735
TT (s)	16.54 ± 1.26	16.70 (15.50–17.55)	16.30 (15.38–17.68)	0.892
INR	0.97 ± 0.07	0.99 (0.92–1.00)	0.99 (0.94–1.02)	0.416

Significant values are in bold.

### Correlations of coagulation markers with high-risk PCa

In the univariate analysis, FIB (OR = 7.518, 95% CI 3.998–14.138, *p* < 0.001) and DD (OR = 12.788, 95% CI 3.626–45.107, *p* < 0.001) were identified as statistically significant parameters positively correlated with PCa severity. To eliminate the potential confounding effects of other variables, we conducted a multivariate logistic regression analysis. Notably, both FIB (OR = 6.986, 95% CI 3.405–14.331, *p* < 0.001) and DD (OR = 4.860, 95% CI 1.124–21.010, *p* < 0.05) remained as independent factors associated with high-risk PCa in the multivariate regression analysis (Table 7).

**Table 7:** Univariate and multivariate analyses of coagulation markers and high-risk PCa.

Variables	Univariate			Multivariate		
	EXP (β)	95% CI	*p* value	EXP (β)	95% CI	*p* value
PLT (×10^9^/L)	1.001	0.996–1.006	0.662	0.993	0.984–1.001	0.090
FIB (g/L)	7.518	3.998–14.138	**0.000**	6.986	3.405–14.331	**0.000**
DD (mg/L)	12.788	3.626–45.107	**0.000**	4.860	1.124–21.010	**0.034**
PT (s)	0.991	0.719–1.365	0.955	1.360	0.532–3.478	0.521
PTA (%)	1.010	0.992–1.029	0.287	1.001	0.898–1.117	0.979
APTT (s)	0.996	0.922–1.077	0.924	1.007	0.886–1.145	0.913
TT (s)	0.846	0.703–1.019	0.078	0.926	0.664–1.292	0.652
INR	0.045	0.001–2.074	0.113	0.000	0.000–46.975	0.490

Significant values are in bold.

## Discussion

As molecular biology research on tumors continues to deepen, an increasing amount of evidence suggests that the coagulation system may be involved in mediating the initiation and progression of malignant tumors^17–19^. Our study is the first to comprehensively investigate the association between comprehensive coagulation markers and low- or high-risk PCa, and we have confirmed that the coagulation markers FIB and DD are closely associated with the invasiveness of PCa.

We observed a positive correlation between plasma levels of FIB and DD in patients with PCa and more advanced tumor stages, including T, N, and M stages. Meanwhile, levels of PSA and ISUP grades also increased with higher levels of FIB and DD. Moreover, our study demonstrated that FIB and DD are independent risk factors for high-risk PCa. Elevated plasma levels of FIB and DD may indicate unfavorable clinicopathological features and a poorer prognosis. Therefore, the integration of plasma levels of FIB and DD into the existing PCa risk stratification system may assist clinicians in formulating more reasonable treatment strategies and appropriate follow-up plans.

FIB is a high-molecular-weight soluble glycoprotein consisting of three peptide chains connected by 29 disulfide bonds and having a molecular weight of 340 kDa. It is a critical protein in the body's coagulation process. FIB may promote the progression of PCa through multiple mechanisms. Firstly, FIB can create a protective fibrin shield surrounding tumor cells, enhancing their resistance to endogenous immune mechanisms and providing a stable structural framework for the extracellular matrix of tumor cells. This supports the action of cell factors such as vascular endothelial growth factor and fibroblast growth factor on tumor cells, ultimately promoting tumor proliferation and angiogenesis^20^. Secondly, FIB receptors on tumor cells can facilitate the connection of FIB molecules to the tumor cells, which enhances tumor cell endothelial adhesion in the vascular system of the target organ and promotes tumor metastasis^21^. Additionally, FIB can promote tumor cell adhesion to platelets through β3-integrin-mediated pathways, leading to the formation of PLT-tumor cell aggregates, which may confer protection against immune system recognition and promote tumor cell metastasis^22^. It has been reported that the absence of fibrinogen can considerably diminish the spontaneous metastatic potential of invasive tumor cell lines, including those that disseminate through the blood and lymphatic systems^23^. Prior investigations have demonstrated that plasma FIB levels are independent prognostic factors for overall survival, cancer-specific survival, and progression-free survival among patients diagnosed with PCa^24^. Song et al. recently reported similar findings, demonstrating a significant association between elevated plasma FIB levels and reduced survival rates in PCa patients^25^. These findings collectively indicate a correlation between plasma FIB levels and the prognosis of patients with PCa. Nevertheless, the association between plasma FIB levels and clinicopathological features of PCa remains poorly understood, as only a limited number of studies have investigated this relationship. Previous studies have documented a positive association between plasma FIB levels and PSA levels, T stage, and Gleason score in patients with PCa^26^, consistent with our results. Furthermore, Xie et al. suggested that plasma FIB levels before treatment in PCa patients were positively correlated with bone metastasis burden and that fibrinogen may be a potential predictive factor for high bone metastasis burden in PCa patients^27^. Our findings also support a positive correlation between high plasma FIB levels and distant metastases, which is in line with previous studies. The aforementioned research findings unequivocally establish a substantial correlation between plasma FIB levels and the severity of PCa. However, previous studies examining the relationship between plasma FIB levels and PCa failed to take other coagulation markers into account. In contrast, our study comprehensively investigated the correlation between comprehensive coagulation markers and the clinicopathological features of PCa.

DD is a stable end product resulting from the degradation of cross-linked fibrin and a specific marker of fibrinolysis. Its level is known to increase with hyperactivity of the fibrinolytic system. Prior investigations have demonstrated that DD plasma levels are markedly elevated in individuals with PCa relative to those with benign prostatic hyperplasia^28^. The elevated plasma level of DD is strongly associated with an elevated risk of mortality in patients with PCa, and it may act as an independent risk factor for an unfavorable prognosis in such individuals^29^. Additionally, in another study, it was reported that the plasma level of DD was markedly higher in patients with late-stage PCa compared to those with localized disease^30^. In our investigation, we stratified PCa patients into three groups based on their DD levels and found the percentage of patients with late-stage PCa significantly increased with rising DD levels (from G1 to G3), which is consistent with the aforementioned research findings.

The mechanism underlying the elevation of plasma FIB and DD levels in PCa patients exhibiting unfavorable clinicopathological features remains incompletely elucidated. PCa cells can increase plasma FIB levels by secreting related cytokines or through endogenous synthesis. Furthermore, PCa cells can cause dysfunction in the body's fibrinolysis system, leading to elevated plasma FIB and DD levels^31^. We hypothesize that high-risk PCa cells may have a stronger ability to secrete cytokines and synthesize FIB than cells corresponding to low-intermediate risk PCa. This hypothesis requires further investigation.

In our study, we did not observe any significant association between PLT, APTT, PT, PTA, TT, INR, and the severity of PCa in patients. Moreover, PLT, APTT, PT, PTA, TT, and INR demonstrated no elevation or reduction with escalating FIB and DD levels (Tables 4 and 6).

The activation of PLT is an indispensable prerequisite for enhanced coagulation function. Malignant neoplasms have been shown to induce dysregulation of coagulation function in the body, leading to a rapid increase in the circulating PLT count^32,33^. PLT in the circulation can shield tumor cells from immune system attacks and other pro-apoptotic stimuli and promote tumor growth and metastasis by regulating the tumor microenvironment, releasing growth factors, promoting angiogenesis, and inducing epithelial-mesenchymal transition^34–36^. Thrombocytosis has been reported to have a negative impact on the prognosis of patients with solid tumors. Diminishing the PLT count in circulation can significantly impede tumor growth and metastasis^37^. However, the association between the PLT count and the severity of PCa remains controversial. Sylman et al. reported a significant correlation between increased PLT counts and reduced overall survival in patients with PCa, suggesting that the PLT count could serve as a valuable prognostic indicator for prostate cancer^38^. Nevertheless, in another study, the researchers showed no association between elevated PLT counts and the clinical-pathological characteristics or prognosis of PCa patients, which is consistent with our study findings^39^. The discrepant findings among studies could potentially be attributed to the absence of standardized inclusion and exclusion criteria, differences in the patient populations investigated, as well as variations in the laboratory testing methods and reagents employed.

PT is mainly an indicator of the extrinsic coagulation pathway and the characteristic coagulation cascade, while APTT primarily reflects the intrinsic coagulation pathway and the common pathway of the coagulation cascade. PTA mirrors liver function and the content of coagulation factors in the body^40^. INR standardizes PT values obtained from various laboratories with different reagents, thus enabling uniform medication standards^41^. TT predominantly reflects the transformation of fibrinogen into fibrin^42^. It has been previously reported that APTT and PT are independent prognostic factors for 3-year recurrence-free survival in breast cancer patients^43^. A significant association between TT and 5-year survival has been observed in patients with esophageal squamous cell carcinoma, indicating that TT is a valuable independent prognostic factor in these patients^44^. Furthermore, existing evidence has demonstrated a noteworthy correlation between increased INR and unfavorable survival rates among individuals diagnosed with epithelial ovarian cancer^45^. However, to our knowledge, no studies have currently explored the correlation between APTT, PT, PTA, TT, INR, and the clinicopathological features of PCa. Thus, our study is the first to explore the potential correlation between these coagulation indicators and the severity of PCa.

This study has some inherent limitations that require acknowledgment. Firstly, the study was conducted at a single data center, which may limit the generalizability of the results to other settings. Secondly, the assessment of coagulation parameters was not repeated after the initial evaluation, which may have limited the accuracy of the findings. Additionally, this study did not evaluate the prognostic significance of FIB and DD in relation to PCa. Despite these limitations, we believe that our study is a pioneering effort as it is the first to comprehensively investigate the association between comprehensive coagulation markers and low- or high-risk PCa.

## Conclusions

In conclusion, our study has demonstrated a significant association between the levels of FIB and DD in plasma and the severity of PCa, suggesting that FIB and DD have the potential to serve as valuable biomarkers for PCa risk stratification. Although the exact mechanisms underlying this association remain unclear, our results provide strong evidence for further investigation.

## Data Availability

The datasets generated during and/or analysed during the current study are available from the corresponding author on reasonable request.
